# In silico comparative study of SARS-CoV-2 proteins and antigenic proteins in BCG, OPV, MMR and other vaccines: evidence of a possible putative protective effect

**DOI:** 10.1186/s12859-021-04045-3

**Published:** 2021-03-26

**Authors:** Sondes Haddad-Boubaker, Houcemeddine Othman, Rabeb Touati, Kaouther Ayouni, Marwa Lakhal, Imen Ben Mustapha, Kais Ghedira, Maher Kharrat, Henda Triki

**Affiliations:** 1 Laboratory of Clinical Virology, WHO Regional Reference Laboratory for Poliomyelitis and Measles for the EMR, Institut Pasteur de Tunis, University of Tunis El Manar, 13 place Pasteur, BP74 1002 le Belvédère, Tunis, Tunisia; 2 LR20IPT10 Laboratory of Virus, Host and Vectors, Institut Pasteur de Tunis, University of Tunis El Manar, Tunis, Tunisia; 3 Sydney Brenner Institute for Molecular Bioscience, University of the Witwatersrand, Johannesburg, South Africa; 4 LR99ES10 Human Genetics Laboratory, Faculty of Medicine of Tunis (FMT), University of Tunis El Manar, Tunis, Tunisia; 5 LR11-IPT02 Laboratory of Transmission, Control and Immunobiology of Infections, Institut Pasteur de Tunis, University of Tunis El Manar, Tunis, Tunisia; 6 LR16IPT09 Laboratory of Biomathematics, Biomathematics and Biostatistics, Institut Pasteur de Tunis, University of Tunis El Manar, Tunis, Tunisia

**Keywords:** SARS-CoV-2, BCG, OPV, MMR, Immunogenicity, Vaccine, Putative protection

## Abstract

**Background:**

Coronavirus Disease 2019 (COVID-19) is a viral pandemic disease that may induce severe pneumonia in humans. In this paper, we investigated the putative implication of 12 vaccines, including BCG, OPV and MMR in the protection against COVID-19. Sequences of the main antigenic proteins in the investigated vaccines and SARS-CoV-2 proteins were compared to identify similar patterns. The immunogenic effect of identified segments was, then, assessed using a combination of structural and antigenicity prediction tools.

**Results:**

A total of 14 highly similar segments were identified in the investigated vaccines. Structural and antigenicity prediction analysis showed that, among the identified patterns, three segments in Hepatitis B, Tetanus, and Measles proteins presented antigenic properties that can induce putative protective effect against COVID-19.

**Conclusions:**

Our results suggest a possible protective effect of HBV, Tetanus and Measles vaccines against COVID-19, which may explain the variation of the disease severity among regions.

**Supplementary Information:**

The online version contains supplementary material available at 10.1186/s12859-021-04045-3.

## Background

Since December 2019, an emerging coronavirus called Severe Acute Respiratory Syndrome Coronavirus 2 (SARS-CoV-2) has been spreading worldwide. This novel pathogen is responsible for the Coronavirus Disease 2019 (COVID-19), causing a worldwide pandemic, as declared by the World Health Organization (WHO) in March 2020 [1]. So far, SARS-CoV-2 has been responsible for more than 91 million confirmed cases and more than a million fatalities (from December 8, 2019 to January 15, 2021) [2].

SARS-CoV-2 is an enveloped, positive-sense single-stranded RNA virus. It belongs to the family of *Coronaviridae,* the subfamily of O*rthocoronavirinae* and the genus Betacoronavirus [3]. The viral genome is composed of approximately 29,903 nucleotides; it contains two untranslated regions (5′ and 3′) and eleven Open Reading Frames (ORF) encoding twelve proteins including the Spike (S) and the Nucleocapsid (N) proteins identified as the main antigenic proteins [4, 5].

The number of COVID-19 patients and death cases varied from a region to another [6]. In many countries, an important number of confirmed cases were reported, such as in the United States of America (USA), Brazil, India and Russia causing [6–9]. For instance, in the USA, more than eight million confirmed cases and two hundred thousand deaths were recorded until the 18^th^ of October 2020 [6, 9]. However, in other regions, such as Madagascar, Sierra Leone, Nicaragua and Uruguay, the number of cases seems to be limited [6, 10, 11] and no more than three hundred deaths were notified [6]. The variation in the number of deaths and infections among countries can be explained by different factors, such as health infrastructure, mitigation strategies and also cultural behavior [12, 13]. The immunological background of the population, mainly due to the vaccination strategies used in those countries was also suggested [13–15]. Indeed, it was previously demonstrated that administration of attenuated vaccines such as OPV (Oral Poliovirus Vaccine), MMR (Measles, Mumps and Rubella vaccines) and BCG (Bacillus Calmette-Guérin) vaccines could improve the innate immune response to fight different pathogens [13–15]. Furthermore, it was suggested that the adoption of a universal and long-standing BCG policy may have a protective effect against COVID-19 [13]. However, and to date, no comprehensive fundamental evidence showed a relationship between regular vaccination and the acquisition of immunity to SARS-CoV-2. In recent epidemiological study, based on a large cohort of patients, no links between the administration of BCG vaccine and COVID-19 severity was found [16]; but, after refining the epidemiological study, a strong correlation was reported [17]. The protective potential of the MMR vaccine was also investigated based on bioinformatic analysis of the S protein [18]. However, no similarity with the crystal structure of S protein, in the Wuhan-Hu-1 isolate (MN908947.3), has been reported [18].

In this paper, we investigated the putative protective role against COVID-19 of three live attenuated vaccines (BCG, OPV and MMR) and nine inactivated vaccines (Tetanus, *Corynebacterium diphtheriae, Bordetella pertussis,* Hepatitis B, Hepatitis A, *Haemophilus influenzae type B* (Hib) and *Streptococcus pneumoniae* vaccines (PCV10)). Our aim was to identify similar amino-acid patterns in all SARS-CoV-2 proteins and the main antigenic proteins of the above-mentioned vaccines and to predict their immunogenicity, using a combination of bioinformatic tools. The in silico identified patterns may be the target of cross-reactive antibodies against their specific pathogen and SARS-CoV-2 and/or may induce cellular immunity.

## Results

### Amino acid sequence alignment and hot spot analysis

The global amino acid identity between the main antigenic protein of investigated vaccines and SARS-CoV-2 proteins does not exceed 63%. For structural proteins, it varied between 21 and 55% (identity levels for the S and M proteins respectively with the Polyprotein E1/E2 of the Rubella virus and the HAV VP1 protein). For non-structural proteins, identity levels varied between 21 and 63% (identity rates of ORF1a and ORF3a proteins respectively with HBsAg-adr protein of Hepatitis B virus and Tetanus Toxin protein) (Additional file 1).

Similar segments with main vaccine antigenic proteins were identified along with structural and non-structural proteins of SARS-CoV-2. The majority were shorter than five consecutive amino-acids for all SARS-CoV-2 proteins (Additional file 2). Nevertheless, a total of twelve patterns of six to eight similar consecutive amino-acids were identified in comparison with the main antigenic proteins of Poliovirus, Measles, *Streptococcus pneumoniae*, Tetanus, Mumps, Hepatitis B, Hib and BCG vaccines (Table1). Two similar segments were identified through comparison of Poliovirus, Measles, PCV10 and Hib proteins and SARS-CoV-2 structural proteins (S and N) and also non-structural proteins (ORF 1a, ORF 6 and ORF 8). In contrast, Tetanus, Mumps, Hepatitis B and BCG antigenic proteins showed no more than one similar segment with SARS-CoV-2 proteins (Table1). Among the described peptides, seven were similar to others in the S protein of SARS-CoV-2 and were identified in the antigenic proteins in poliovirus Sabin 3, *S pneumoniae*, tetanus, Mumps, Hepatitis B and Hib vaccines. The pattern’s length varied between six and seven amino acids. Also, one peptide of eight amino acids (GTSPARMA), detected in the Poliovirus VP1 sequence, matched with the N protein of the SARS-CoV-2.

**Table 1:** Description of similar patterns of more than five amino-acids obtained in vaccine antigenic proteins and SARS-CoV-2 proteins

Vaccine	No of similar segment	Vaccine protein			SARS-CoV-2 protein		
		Designation	Segment	Position	Designation	Segment	Position
Poliovirus	2	VP1 protein (Sabin 1)	GTAPARIS	188–195	N	GTSPARMA	203–211
		VP1 protein (Sabin 3)	LDPLSE	289–295	S	LDPLSE	293–299
Measles	2	Fusion protein	QECLRG	359–364	ORF6	QECVRG	21–27
			IQVGSRR	433–440	ORF8	IRVGARK	47–54
*Streptococcus pneumoniae*	2	Capsular polysaccharide biosynthesis protein (serotype19F, 18C, 14, 7, 4, 1)	IGFLAGVI	182–190	S	LGFIAGLI	1218–1226
		Capsular polysaccharide biosynthesis protein (serotype19F, 18C, 14, 5)	SSVAFA	33–39	S	NSVAYS	703–708
*Hib*	2	Capsular polysaccharide biosynthesis protein	KNINDS	210–215	S	KNLNES	1191–1196
			FILNKKI	73–79	ORF1a	FLLNKEM	3183–3189
BCG	1	Immunogenic protein MPB64	IFMLVT	5–11	E	VFLLVT	25–31
Tetanus	1	Toxin protein	NILMQY	84–90	S	NLLLQY	751–756
Mumps virus	1	Fusion protein	DISTEL	448–454	S	DISTEI	467–473
Hepatitis B	1	HBs Ag-adr	PGTSTTS	111–117	S	PGTNTSN	600–606

We also identified two discontinuous patterns of 10 amino-acids each, DISGFNSSVI and MSLSLLDLYL, in the tetanus toxin and the hemagglutinin Measles virus proteins which had 90% and 80% similarity with matching segments, DISGINASVV (1168-1177aa), IELSLIDFYL (2-11aa), in the S and ORF7b proteins of SARS-CoV-2 respectively.

### Immunogenicity prediction

First, we focused on characterizing the immunogenicity of the matching sequences with S and N proteins for their involvement in modulating the immune response of the host [19, 20].

Regarding the pattern GTAPARIS matching with N protein sequence (GTSPARMA), it did not map to the structure of the N protein from SARS-CoV-2. Moreover, no significant match with CMH-I predicted epitope was distinguished. The prediction of the B-cell epitope using the N protein sequence showed a potential antigenic peptide of 51 amino acids (165–216) that harbors the pattern GTSPARMA identified from our similarity search.

**Fig. 1:**
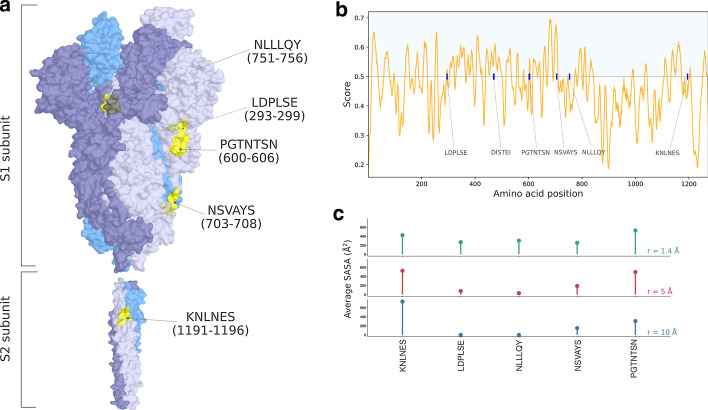
Structural mapping in S protein of the segments that match the antigenic proteins from different pathogens. **A** The location of the segments on the structure is marked by yellow patches. Different chains are represented in different colors. The S1 and S2 subunits have been solved independently. **B** B-cell epitope prediction from the sequence of SARS-CoV-2 protein. The sequences identified from the similarity analysis are marked in blue. Segments in which amino acid scores are above 0.5 are putative epitope sites. **C** Cumulative SASA measures for each of the putative antigenic sites calculated using different probe radii

Among the seven patterns identified in the SARS-CoV-2 S protein, four segments (LDPLSE, NSVAYS, NLLLQY, PGTNTSN) from Polio, PCV10, Tetanus and HBV vaccines, respectively, have been mapped on the structure of the spike protein S1 subunit (Fig. 1A). We were also able to map one other pattern, KNLNE, on the structure of the six-helical bundle fusion core solved independently (S2 subunit) from the rest of the ectodomain. The two other patterns (LGFIAGLI, and DISTEI) were not solved by the electron density map from the Cryo-EM structures. Among the five retained patterns, the segments PGTNTSN and LGFIAGLI showed a putative interaction with one of the MHC-I receptors predicted by IEDB analysis resource NetMHCpan. Furthermore, the prediction for these two peptides showed a weak peptide score of 0.07 and 0.02, respectively (0 indicates no MHC-I capacity, and 1 indicates a high probability). The segment PGTNTSN, existing in the Hbs Ag of Hepatitis B virus adr strain, is located in a turn region.

On the other hand, the prediction of epitopes for B-cell response using Bepipred 2.0 from the IEDB analysis resource showed the implication of four putative patterns from the total set of the seven segments, namely LDPLSE, NSVAYS, DISTEI and PGTNTSN. These segments match the predicted epitopes LDPL, YTMSLGAENSVAYSNN, NLDSKVGGNYNYLYRLFRKSNLKPFERDISTEIYQAGSTPCNGVEGFNCYFPLQSYGFQPTN and TNTSN (Fig. 1B). The sequence KNLNES does not fall in a putative B-cell epitope region.

We also calculated the Solvent Accessible Surface Area (SASA) using different probe radii to allow better insight into the possible interaction of antibody Complementarity-Determining Regions (CDRs) to the predicted epitopes (Fig. 1C). Our results show that exposure to both water molecules and the antibody paratope is only preserved for the segment "PGTNTSN". Consequently, the SASA values at probe radii of 1.4 Å, 5 Å, and 10 Å are 528.69 Å^2^, 497.6 Å^2^, and 305.38 Å^2^, respectively.

**Fig. 2:**
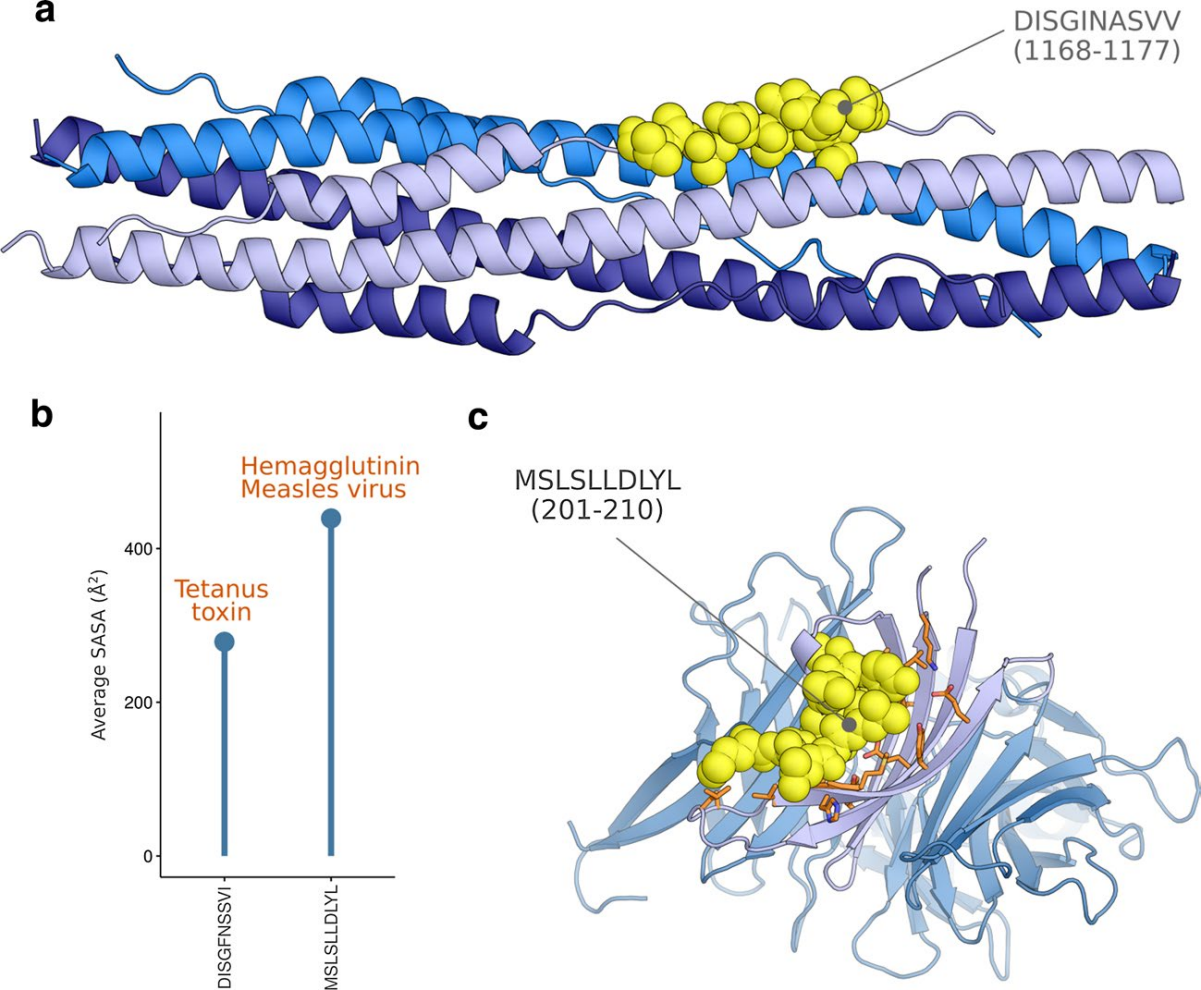
Structural properties of CMH-I putative epitopes resembling segments from Tetanus toxin protein and chain A of the Measles hemagglutinin Protein. **A** Location of DISGINASVV on the structure of the six-helical bundle fusion core from the spike protein. **B** Calculation of SASA for the vaccines DISGFNSSVI and MSLSLLDLYL segment using a probe of 1.4 Å. **C** Crystal structure of the Measles virus hemagglutinin [22]. The peptide (in yellow) shows putative T-cell immunogenicity with the interaction pocket residues (light purple)

Second, we focused on a list of hits that belonged to the investigated vaccine sequences and that match any of the other proteins of SARS-CoV-2. All the patterns have been explored for their antigenic potential using IEDB Bepipred and IEDB NetMHCpan methods. None of the investigated patterns showed a significant putative B-cell antibody binding property. Discontinuous patterns with more than ten residues were discarded from the analysis as they showed low levels of similarity. Consequently, we have retained two segments from Tetanus toxin protein (DISGFNSSVI) and chain A hemagglutinin protein of the Measles virus (MSLSLLDLYL) that significantly matched SARS-CoV-2 Spike and ORF7b proteins, respectively. The segment DISGINASVV of the S protein (Fig. 2A) showed a putative interaction with the MHC-I receptor encoded by one of the corresponding HLA alleles. DISGINASVV and corresponding matching segment DISGFNSSVI showed high peptide scores of 0.88 and 0.76 for the SARS-CoV-2 S and the tetanus toxin proteins, respectively. The segment DISGINASVV is part of the six-helical bundle fusion core of the spike protein. It belongs to the HR2 domain as a random coil structure [21]. The peptide shows an extended conformation within its native environment stabilized by the residues of a small groove formed between two HR1 parallel helices from different monomers. The SASA value for DISGINASVV peptide is 504.88 Å^2^. In contrast, its matching sequence from Tetanus toxin DISGFNSSVI corresponds to a SASA value of 243.3 Å^2^ (Fig. 2B) and the Bepipred tool shows only a partial implication of the sub-string "DISGI" as an epitope in the context of B-cell response.

Regarding the ORF7b and Measles hemagglutinin proteins, the identified similar segments overlap significantly with regions of putative T-cell antigenicity. The matching segment of the Measles hemagglutinin protein (Fig. 2C) corresponded to a random coil segment (MSLS) spanned by an alpha helix of six residues (LLDLYL) in the crystal structure of the hemagglutinin [22]. The segment also interacts with a large pocket formed mainly by four strands of a beta-sheet containing many aromatic amino acids. The pocket is similar to the groove of the MHC-I molecule (Fig. 2C and Additional file 3). Moreover, MSLSLLDLYL corresponds to a SASA measured at 439.19 Å^2^ (Fig. 2B). The NetMHCpan tool predicted an antigenicity score of 0.18 for the MSLSLLDLYL segment using the sequence of ORF7b. We also noticed that the matching segment of the Measles hemagglutinin Protein, i.e. “IELSLIDFYL” is represented by a substring “IELSLIDFY” that shows the highest antigenicity score of 0.59 among all the predicted epitopes.

## Discussion

In this study, we investigated the potential protective effect against COVID-19 induced by regularly used vaccines. In the aim to assess their possible implication of in the immune response against SARS-CoV-2, we used a combination of sequence similarity analysis, structural and antigenicity prediction tools to evaluate main antigenic proteins in twelve commonly used vaccines including BCG, OPV and MMR vaccines.

In our study, we identified of similar patterns and found that most of the detected segments were shorter than five amino acids; therefore, they could not constitute a putative T-cell or B-cell epitopes [23–25].

Nevertheless, twelve patterns of six to eight amino-acids were found and further investigated. We think that PGTNTSN is the most putative to bind to endogenous antibodies among the four patterns that have been identified by the B-cell epitope prediction tool. Segments of less than 5 amino acids such as the LDPL, a substring of the LDPLSE, are rarely responsible for inducing humoral immunity response [25]. Moreover, NSVAYS and DISTEI segments are shorter with 10 and 56 amino acids less than the matching predicted epitopes using the entire sequence of the spike protein from SARS-CoV-2. In such a case, the sequence length would be a constraining factor in reproducing the immunological properties for the studied vaccines. That also applies to GTSPARMA segment which is a substring of 51 amino acid putative epitope from the N protein. On the other hand, the PGTNTSN segment of SARS-CoV-2 matches with the predicted epitope TNTSN which is only shorter by two amino acids, compared to both patterns identified for SARS-CoV-2 and its matching segment on HBs Ag-adr.

The pattern PGTNTSN detected in HBsAg of Hepatitis B virus corresponded to an exposed site in the S protein and showed the highest values of accessible surface area compared to the segments identified in the S1 subunit. Additionally, the accessibility of PGTNTSN to the probing spheres mimicking the CDRs antibodies supports its implication in the B-cell mediated response. Thus, its structural properties were consistent with its putative neutralizing capacity. Naturally, the antibodies would be able to recognize the targeted epitope on the whole assembled structure of the virus, and therefore, the epitope must be accessible at the surface of the spike protein. On the other hand, in their recent attempt to establish the antigenicity map of SARS-CoV-2, Zhang et al. have found that a segment called IDh spanning residues 522–646 induces a positive B-cells reaction in sera of convalescent COVID-19 patients [20]. The pattern PGTNTSN was included in the IDh epitope and we were able to identify strong prediction metrics using the IEDB Bepipred tool. Therefore, the induced immunological reaction by this segment would be a humoral response. Furthermore, our results were in agreement with the work published by Tajiri et al. [26] who showed that two regions of HBsAg (residues 104–123 and 108–123) containing the epitope matching the PGTNTSN segment of SARS-CoV-2, were able to bind with two human monoclonal antibodies. This highlighted the immunogenic capability of these segments. There have been concerns about the antibody-dependent enhancement (ADE) of the SARS-CoV-2 infection due to the possible activation of effector functions [27]. The antibody repertoire is thought to be the main culprit for such an effect [28]. However, its magnitude still unknown and recent evidence suggests a non-significant or unclear contribution in enhancing the infectivity of SARS-CoV-2. For instance, the expression of Fcγ receptors through which the effector functions are triggered seems to be very low in alveolar, bronchial, and nasal-cavity epithelial cells (*idem*). Moreover, it is difficult to distinguish the contribution of the antibody-dependent enhancement of the infection from a severity due to other factors. Recently, in a detailed review, Arvin et al. have stated that *current clinical experience is insufficient to implicate a role for ADE of disease, or immune enhancement by any other mechanism, in the severity of COVID-19* [28].

The segment PGTNTSN is located away from the RBD interaction site to ACE2, separated by an approximate distance of 75 Å. However, the putative antigen, is very close to the fusion peptide SFIEDLLFNKV (residues 816–826 on the PDB structure 7BYR) located at an approximate distance of 35 Å. Moreover, the same region includes the S21P2 segment that has been identified as the epitope for antibodies targeting protein S and enabling the neutralization of the SARS-CoV-2 pseudovirus infection [28]. Therefore, it would be possible to have the same scenario for the PGTNTSN predicted epitope. Furthermore, the location of the PGTNTSN segment overlaps with a putative interaction surface with TMPRSS2 which would impact the cleavage of S1/S2 and S2 sites required for the priming of the S protein [29, 30].

On the other hand, and considering the S protein conservation, which is constantly facing a selective pressure from the immune system, several studies demonstrated the existence of highly conserved domains in the S protein such as “SD2.1” (amino acids 589–605) which matches with the ‘PGTNTSN’ segment (600–606) [31–33]*.* Still, only, randomized controlled trials might provide evidence of induced protective effect against COVID-19. In many countries, the HBV vaccine is commonly recommended or mandatory for healthcare and wet lab workers. Therefore, it would be interesting to investigate the prevalence of SARS-CoV-2 and clinical manifestations of COVID-19 among HBV vaccinated health workers.

Interestingly, our analysis showed the presence of two segments of ten amino acids from the Tetanus toxin protein and the chain A of the Measles hemagglutinin protein, similar to others located in the S and ORF7b proteins of SARS-CoV-2. The segment DISGINASVV, matching with the toxin tetanus protein has been previously described to be part of an antigenic peptide in the S protein of SARS-CoV-2 [34]. Trigueiro-Louro et al. performed a structure-based strategy targeting highly conserved regions in the Spike domains and demonstrated that the domain “CD-HR2.1” (amino acids 1112–1232), that matches with the regions DISGINASVV, is a “highly conserved druggable regions” [14]. Regarding the segment matching with the ORF7b protein, which may have an accessory function and whose role is yet to be determined [35], we could not exclude its possible immunogenic role. On the other hand, we have also recorded a significant global identity level between the Measles fusion and hemagglutinin proteins and SARS-CoV-2 spike, envelope and matrix proteins (45–50%) (suppl mat. 1). Furthermore, another study using other Measles and Rubella sequences, different from Edmonston Measles and Wistar RA 27/3 Rubella vaccine strains, revealed similarity between the N terminal region of SARS-COV-2 Spike protein and the Fusion protein of Measles virus as well as the envelope protein of Rubella virus. Still, no similarity was obtained with the crystal structure [18]. It was previously demonstrated that live attenuated vaccines such as OPV, BCG and MMR could improve the innate immune response to other pathogens [36]. These non-specific effects of live vaccines involved the trained immunity which refers to the memory-like characteristics of innate immune cells [37]. Indeed, following exposure to a primary stimulus like a vaccine or a microbial component, innate immune cells, especially monocytes and NK-cells, undergo epigenetic reprogramming that subsequently regulates cytokine production and cell metabolism and it collectively enhances responsiveness to an unrelated secondary stimulus. In this line, observational studies reported a decrease in hospitalization rate and overall mortality among children immunized with live attenuated vaccines [14]. Furthermore, pediatric populations seem to be less vulnerable to COVID-19, especially in low and middle- income countries [14, 38, 39]. The long-term use of an attenuated vaccine, with high coverage rate, could, partially, explain the low symptomatic infection rate among children. Thus, epidemiological studies targeting a largely vaccinated population can help in assessing the protective effect of the MMR vaccine against COVID-19.

## Conclusions

Since December 2019, the novel Coronavirus, SARS-CoV-2, spread all around the word causing a worldwide pandemic, and more than 91 million confirmed cases and a million fatalities.

Using an in silico strategy, this study suggests a possible protective effect of HBV, Tetanus and Measles vaccines against SARS-CoV-2 which should be confirmed by extensive epidemiological studies targeting large populations. This possible cross-protection may explain the variation of the disease severity among countries.

## Material and methods

### Investigated vaccines and sequences

Our study focused on twelve vaccines including live attenuated (BCG, OPV, MMR vaccines) and inactivated ones (*Tetanus*, *Corynebacterium diphtheriae, Bordetella pertussis,* Hepatitis B, Hepatitis A, *Haemophilus influenzae type B* (Hib) and *Streptococcus pneumoniae* vaccines (PCV10) (Table 2).

**Table 2:** vaccines and corresponding antigenic proteins investigated in this study

Vaccine	Protein	Accession N°	Reference
*Tetanus*	Toxin protein	AAA23282.1	[40]
*Corynebacterium diphtheriae*	Toxin protein	CAA00374.1	[40]
Hepatitis B	HBsAg-adw2	AAW65557.1	[41]
	HBsAg-adr	AAW65588.1	
*Bordetella pertussis*	Toxin protein	AQW64178.1	[40]
Measles	Hemagglutinin protein	AAF85705.1	
	Fusion protein	AAF85704.1	[42]
Rubella	Polyprotein E1/E2	ACN50046.1	[42]
Mumps	Fusion protein	ACN50030.1	
	Hemagglutinin/neuraminidase protein	ACN50032.1	[42]
Hepatitis A	VP1 protein	AAA45466.1	
	VP3 protein	AAA45466.1	[43, 44]
Bacillus Calmette-Guérin (BCG)	Immunogenic protein MPB83	BAA11027.1	
	Immunogenic protein MPB70	BAA07402.1	[45–47]
	Immunogenic protein MPB64	AIC33023.1	
*Hemophilus influenzae* serotype B (Hib)	Capsulation protein	CWW30252.1	[40]
	Capsular polysaccharide biosynthesis protein	WP_015702013.1	
Poliovirus	VP1 protein (Sabin 1 strain)	AAL89597.1	[48]
	VP1 protein (Sabin 2 strain)	AAL92486.1	
	VP1 protein (Sabin 3 strain)	AAL89592.1	
*Streptococcus pneumoniae* (PCV10)	Capsular polysaccharide biosynthesis protein [serotype 19F]	AEO88919.1	[49]
	Capsular polysaccharide biosynthesis protein [serotype 23F]	AAC69522.1	
	Capsular polysaccharide biosynthesis protein [serotype 18C]	CAI33577.1	
	Capsular polysaccharide biosynthesis protein [serotype 14]	CAI33319.1	
	Capsular polysaccharide biosynthesis protein [serotype 9 V]	CAI33023.1	
	Capsular polysaccharide biosynthesis protein [serotype 7F]	CAI32924.1	
	Capsular polysaccharide biosynthesis protein [serotype 6B]	AAK20683.1	
	Capsular polysaccharide biosynthesis protein [serotype 5]	CAI32793.1	
	Capsular polysaccharide biosynthesis protein [serotype 1]	COS99248.1	
	Capsular polysaccharide biosynthesis protein [serotype 4]	AAK20668.1	

The full amino-acid sequences of the main antigenic proteins (n = 30) corresponding to the 12 investigated vaccines were obtained from NCBI Genbank database (https://www.ncbi.nlm.nih.gov). Accession numbers are listed in Table 2. In addition, the amino-acid sequences of the structural proteins (Spike (S), Envelope (E), Membrane glycoprotein (M), Nucleocapsid (N) and non-structural proteins (ORF1ab, ORF1a, ORF3a, ORF6, ORF7a, ORF7ab, ORF8 and ORF10) of SARS-CoV-2 Wuhan reference strain (NC_045512) were obtained from NCBI.

### Amino acid sequence alignment and hot spot analysis

Identification of similar segments, including identical amino-acids and/or similar amino-acids (with similar biochemical properties), was assessed using Blastp homology search by querying the protein sequences of SARS-CoV-2 over the set of antigenic sequences of the vaccines [50]. Blast 2 sequences tool was used with an Expect threshold (E-value) of 10, in order to see shorter alignments, according to the stochastic model of Karlin and Altschul (1990) [51]. Pairwise alignments obtained from Blastp were explored and analyzed using BioEdit software, version 7.2.5 (http://www.mybiosoftware.com/bioedit-7-0-9-biological-sequence-alignment-editor.html).

### Structural analysis and antigenicity prediction

The structure of the SARS-CoV-2 spike protein was obtained from PDB entries 7BYR [52] and 6LXT [21] corresponding to the structure of S1 and S2 subunits respectively. Both structures showed a respective sequence identity of 99.6 and 100% compared to the reference sequence of the S protein from the Wuhan-Hu-1 isolate of SARS-CoV-2 (accession number YP_009724390.1 for the spike protein). The segments matching one of the sequences of S and N proteins were mapped on the structure. The Solvent Accessible Surface Area (SASA) per residue was calculated using freesasa [52]. The B-cell and T-cell epitope predictions were conducted using IEDB analysis resource Bepipred 2.0 [29] and the IEDB analysis resource NetMHCpan [53] methods by uploading the primary structure of SARS-CoV-2 protein; considering all the possible human HLA alleles for MHC class I. These correspond to HLA genes A, B, C, E, and G and cover 134 alleles from different allele groups. A list of these alleles is provided in Additional file 4. The length of the predicted peptides was set to a default value of 8–11 residues, with respect to the proteasomal processing mechanism [54]. A pattern is retained if it shows a good quality local alignment with no indels and no more than two successive dissimilar residues. The matching pattern of the query has to show significant antigenicity prediction, at least with one of the methods, IEDB Bepipred or IEDB NetMHCpan. A cutoff of peptide score no less than 0.1 was used. At this level, the sensitivity and specificity values would be above 0.9, according to the evaluation by Jutz et al.[55]. For IEDB Bepipred, a putative epitope has to show a score above 0.5 for all its constructing amino acids.

The Solvent Accessible Surface Area (SASA) was calculated residue wise. Three probing radii were used including one that mimics the solvent molecules (1.4 Å) and two other (5 and 10 Å) to access the accessibility of the Antibody Complementarity-Determining region (CDR) to the putative B-cell epitope [56].

## Supplementary Information

**Additional file 1:** Global amino-acid identities between structural protein sequences of SARS-CoV-2 and main antigenic proteins of investigated vaccines.

**Additional file 2:** Similar patterns identified between SARS-CoV-2 proteins and antigenic proteins in investigated vaccines.

**Additional file 3:** Structure of MHC class I heavy chain in complex with Vesicular stomatitis virus nucleoprotein (PDB code 2VAA) [57]. The binding groove floor is composed of 5 strands beta-sheet, resembling the stabilizing beta-sheet from of MSLSLLDLYL peptide within the Measles hemagglutinin Protein.

**Additional file 4:** List of the HLA alleles used for the prediction of CMH-I binding using IEDB analysis resource.

## Data Availability

All data generated or analyzed as part of this study are included in this published article and its supplementary information files. Accession numbers of sequences used in this study are indicated in Table2, in the Material and Methods section of the article. All data generated are available in a public repository https://figshare.com/articles/dataset/Comparative_study_of_SARS-CoV-2_proteins_and_antigenic_proteins_in_BCG_OPV_MMR_and_other_vaccines_evidence_of_possible_putative_protective_effect/13220762
