# Supplementary material for: In silico comparative study of SARS-CoV-2 proteins and antigenic proteins in BCG, OPV, MMR and other vaccines: evidence of a possible putative protective effect
Source: BMC Bioinformatics. 2021 Mar 26;22:163. doi: 10.1186/s12859-021-04045-3 (PMC7995392; doi:10.1186/s12859-021-04045-3)
Supplement: Supplementary file 1 — Additional file 1: Global amino-acid identities between structural protein sequences of SARS-CoV-2 and main antigenic proteins of investigated vaccines. [file 12859_2021_4045_MOESM1_ESM.docx]

**Title:**

***In silico* comparative study of** **SARS-CoV-2 proteins and antigenic proteins in BCG, OPV, MMR and other vaccines: evidence of possible putative protective effect**

**Authors:** Sondes Haddad-Boubaker^1,2^, Houcemeddine Othman^3^, Rabeb Touati^4^, Kaouther Ayouni^1, 2^, Marwa Lakhal^4^, Imen Ben Mustapha^5^, Kais Ghedira^6^, Maher Kharrat^4^ and Henda Triki^1,2^

1 Laboratory of Clinical Virology, WHO Regional Reference Laboratory for Poliomyelitis and Measles for EMRO region, Institut Pasteur de Tunis, University of Tunis El Manar, Tunisia.

2. Laboratory of Virus, Host and vectors, Institut Pasteur de Tunis, University of Tunis El Manar, Tunisia.

3 Sydney Brenner Institute for Molecular Bioscience, University of the Witwatersrand, Johannesburg, South Africa.

4 LR99ES10 Human Genetics Laboratory, Faculty of Medicine of Tunis (FMT), University of Tunis El Manar, Tunisia.

5. Laboratory of Transmission, Control and Immunobiology of Infections. Institut Pasteur de Tunis, University of Tunis El Manar, Tunisia

6. Laboratory of Biomathematics, Biomathematics and Biostatistics. Institut Pasteur de Tunis, University of Tunis El Manar, Tunisia.

**Supplementary material 1: Global amino-acid identities between Sars-CoV-2 protein sequences and main antigenic proteins of investigated vaccines.**

| **Vaccine** | **Protein** | **amino-acid Identity (%)** | | | | | | | | | |  |  |
| --- | --- | --- | --- | --- | --- | --- | --- | --- | --- | --- | --- | --- | --- |
|  |  | **Spike** | **Envelope** | **Glycoprotein**  **Membrane** | **Nucleocapsid** | **ORF1a** | **ORF1ab** | **ORF3a** | **ORF6** | **ORF7a** | **ORF7ab** | **ORF8** | **ORF10** |
| **1** | Toxin protein | 35.29 | - | - | 29.27 | - | - | 63.64 | 27.59 | - | 26.32 | - | - |
| **2** | Toxin protein | 45.00 | - | - | - | - | - | 22.16 | 38.89 | 33,33 | - | - | - |
| **3** | HBsAg-adw2 | 55.56 | - | 33.33 | 28.57 | 29.55 | 21.36 | 30.30 | - | 36 | - | 25.71 | - |
|  | HBsAg-adr | 50.00 | - | 30 | 28.57 | 21.36 | 21.36 | 30.30 | - | 31,35 | 34.48 | 25.71 | - |
| **4** | Toxin protein | 23.26 | - | 45.45 | - | 35.29 | 26.13 | - | - | 28.57 | - | 27.27 | - |
| **5** | Hemagglutinin protein | 23.75 | 45.83 | 50 | 35.71 | 23.42 | - | - | - | - | 60.00 | 31.82 | - |
|  | Fusion protein | 45.45 | - | 37.50 | - | 29.55 | 29.55 | - | 32.00 | 43.48 | - | 25.53 | - |
| **6** | Rubella polyprotein E1/E2 | 21.80 | - | 40.00 | - | 23.40 | 34.62 | 27.59 | - | - | 38.46 | 30.77 | - |
| **7** | Fusion protein | 31.43 | - | 37.50 | - | 27.27 | 27.27 | 24.32 | - | 40 | - | 40.00 | - |
|  | Hemagglutinin/neuraminidase protein | 27.14 | - | 26.47 | - | 33.33 | 33.33 | 30 | 27.27 | 42.86 | - | 60.00 | - |
| **8** | VP1 protein | 34.29 | - | 55.56 | 52.94 | 22.50 | 22.50 | - | - | 20.41 | 27.78 | - | - |
|  | VP3 protein | 50.00 | - | 42.86 | - | 26.67 | 26.15 | 22.50 | - | - | - | 25.58 | - |
| **9** | Immunogenic protein MPB83 | 27.27 | - | 41.18 | - | 34.09 | 43.48 | 27.78 | - | 27,27 | - | - | - |
|  | Immunogenic protein MPB70 | 52.17 | - | 41.18 | - | 38.89 | - | 26.92 | - | - | - | 25,71 | - |
|  | Immunogenic protein MPB64 | 35.48 | 30.30 | 35.71 | - | 25.00 | 25.00 | - | - | 45.45 | - | - | - |
| **10** | Capsulation protein | 25.00 | - | 55.56 | - | 26.00 | 26.00 | - | - | - | - | 46.15 | - |
|  | Capsular polysaccharide biosynthesis protein | 23.16 | - | - | 36.84 | 27.50 | 29.41 | - | - | 26.09 | - | 46.15 | - |
| **11** | VP1 protein (Sabin 1 strain) | 34.48 | - | - | - | 21.67 | 33.33 | - | - | - | - | 46.15 | - |
|  | VP1 protein (Sabin 2 strain) | 42.11 | - | - | - | 33.33 | 33.33 | 62.5 | - | - | - | - | - |
|  | VP1 protein (Sabin 3 strain) | 26.67 | - | - | - | 33.33 | 33.33 | 60.00 | 57.14 | 58.33 | - | - | - |
| **12** | Capsular polysaccharide biosynthesis protein [serotype 19F] | 23.15 | - | - | 33.33 | - | - | - | - | - | - | - | - |
|  | Capsular polysaccharide biosynthesis protein [serotype 23F] | 26.47 | - | - | - | 29.17 | - | - | - | 41.18 | - | - | - |
|  | Capsular polysaccharide biosynthesis protein [serotype 18C] | 35.29 | - | - | 37.04 | 31.43 | - | 20.34 | - | - | - | - | - |
|  | Capsular polysaccharide biosynthesis protein [serotype 14] | 27.38 | - | - | 33.33 | 27.41 | - | 22.03 | - | - | - | - | - |
|  | Capsular polysaccharide biosynthesis protein [serotype 9V] | 43.75 | - | - | - | 34.21 | 34.21 | 30.00 | - | - | - | - | - |
|  | Capsular polysaccharide biosynthesis protein [serotype 7F] | 35.29 | - | - | 37.04 | - | 57.14 | 22.03 | - | - | - | - | - |
|  | Capsular polysaccharide biosynthesis protein [serotype 6B] | 43.75 | - | - | - | 33.33 | 33.33 | 30.00 | - | - | - | - | - |
|  | Capsular polysaccharide biosynthesis protein [serotype 5] | 29.76 | - | - | - | - | 57.14 | 23.73 | - | - | - | - | - |
|  | Capsular polysaccharide biosynthesis protein [serotype 1] | 35.29 | - | - | 37.04 | - | 50.00 | - | - | - | - | - | - |
|  | Capsular polysaccharide biosynthesis protein [serotype 4] | 35.29 | - | 27.27 | 33.33 | - | 57.14 | 30.00 | - | - | - | - | - |

Note: 1:Tetanus; 2: Corynebacterium diphtheriae; 3: Hepatitis B; 4 : Bordetella pertussis; 5: Measles; 6: Rubella ; 7 : Mumps ; 8 : Hepatitis A ; 9: Bacillus Calmette-Guérin (BCG); 10: Hemophilus influenzae type B (Hib); 11: Poliovirus; 12: Streptococcus pneumonia (PCV10); ( - ): no significant Identity.
