# Supplementary material for: In silico comparative study of SARS-CoV-2 proteins and antigenic proteins in BCG, OPV, MMR and other vaccines: evidence of a possible putative protective effect
Source: BMC Bioinformatics. 2021 Mar 26;22:163. doi: 10.1186/s12859-021-04045-3 (PMC7995392; doi:10.1186/s12859-021-04045-3)
Supplement: Supplementary file 2 — Additional file 2: Similar patterns identified between SARS-CoV-2 proteins and antigenic proteins in investigated vaccines. [file 12859_2021_4045_MOESM2_ESM.docx]

**Title:**

***In silico* comparative study of** **SARS-CoV-2 proteins and antigenic proteins in BCG, OPV, MMR and other vaccines: evidence of possible putative protective effect**

**Authors:** Sondes Haddad-Boubaker^1,2^, Houcemeddine Othman^3^, Rabeb Touati^4^, Kaouther Ayouni^1, 2^, Marwa Lakhal^4^, Imen Ben Mustapha^5^, Kais Ghedira^6^, Maher Kharrat^4^ and Henda Triki^1,2^

1 Laboratory of Clinical Virology, WHO Regional Reference Laboratory for Poliomyelitis and Measles for EMRO region, Institut Pasteur de Tunis, University of Tunis El Manar, Tunisia.

2. Laboratory of Virus, Host and vectors, Institut Pasteur de Tunis, University of Tunis El Manar, Tunisia.

3 Sydney Brenner Institute for Molecular Bioscience, University of the Witwatersrand, Johannesburg, South Africa.

4 LR99ES10 Human Genetics Laboratory, Faculty of Medicine of Tunis (FMT), University of Tunis El Manar, Tunisia.

5. Laboratory of Transmission, Control and Immunobiology of Infections. Institut Pasteur de Tunis, University of Tunis El Manar, Tunisia

6. Laboratory of Biomathematics, Biomathematics and Biostatistics. Institut Pasteur de Tunis, University of Tunis El Manar, Tunisia.

**Supplementary material 2** : Similar patterns identified between **Spike protein** of SARS-CoV-2 and antigenic proteins in investigated vaccines: Tetanus (a) , *Corynebacterium diphtheria* (b), Hepatitis B (c1,c2), *Bordetella pertussis* (d), Measles (e1,e2), Rubella (f1), Mumps (g1,g2), Hepatitis A (h1,h2), Bacillus Calmette-Guérin (BCG) (i1,i2,i3), *Haemophilus influenzae* serotype b (Hib) (j1,j2), Poliovirus (k1,k2,k3) and *Streptococcus pneumoniae* (PCV10) (l1,l2,l3,l4,l5,l6,l7,l8,l9,l10). Patterns of six and more similar amino acids are highlighted in yellow.

1. Tetanus toxin protein

### Similar pattern 1:

10 20 30 40

....|....|....|....|....|....|....|....|

**1149 KEELDKYFKNHTSPDVDLG-----DISGINASVV 1177**

**0132 E.DI.VIL.KS.ILNL.INNDIIS....F.S..I 165**

**+ ++ ++ + + +**

### Similar pattern 2:

10 20 30 40

....|....|....|....|....|....|....|....|....|.

**747 TECS****NLLLQYGSFCTQLNRALTGIAVEQDKNTQEVFAQVKQIYKTP 792**

**080 .QSK.I.M..----IKA.SKFI..T-----ELKKLESKINKVFS.. 116**

+ + + + + ++ +++ +++

### Similar pattern 3:

10 20 30 40 50

....|....|....|....|....|....|....|....|....|....|...

**362 VADYSVLYNSASFSTFKCYGVSPTKLNDLCFT------NVYADSFVIRGDEVR 408**

**403 IT..MY.T.AP.YTNG.LNIYYRRLY.G.K.IIKRYTP.NEI....KS..FIK 455**

+ + ++ ++

### Similar pattern 4:

10 20 30

....|....|....|....|....|....|

**417 KIADYNYKLPDDFTGCVIAWNSNNLDSKV 445**

**012 ..I..E..IYSGPDKEQ..DEI...KN.L 40**

+ + +

### Similar pattern 5:

10 20 30

....|....|....|....|....|....|....|...

**007 LLPLVSS----QCVNLTTRTQL--PPAYTNSFTRGVYY 38**

**388 .I.VA..SKDV.LK.I.DYMY.TNA.S...G-KLNI.. 424**

+ + + + +

### Similar pattern 6:

10

....|....|...

**214 RDLPQGFSA 222**

**275 ....DK.N. 283**

+

### Similar pattern 7:

10 20 30 40

....|....|....|....|....|....|....|....|....|.

**997 ITGRLQSLQTYVTQQLIRAAEIRASANLAATKMSECVLGQSKRVDF 1042**

**157 .S.FNS.VI..PDA..VPGINGK.-IH.VNNES..VIVHKAMDIEY 201**

+ + + + + ++ ++ +++

### Similar pattern 8:

10 20 30 40 50

....|....|....|....|....|....|....|....|....|....|....

**811 KPSKRSFIEDLLFNKVTLADAGFIKQYGDCLGDIAARDLICAQKFNGLTVLPPL 864**

**056 RE.S...LVNQMI.EAKKQLLE.DT.SKNI.----MQYIKANS..I.I.E.KK. 105**

+ + + + + + + + +

### Similar pattern 9:

10

....|....|

**409 QIAPGQTGK 417**

**171 .LV..IN.. 179**

+

### Similar pattern 10:

10

....|....|....|

**202 KIYSKHTPINLVRDL 216**

**361 .L.TSYLS.TFL..F 375**

+ + + +

1. *Corynebacterium diphtheria* toxin protein

### Similar pattern 1:

10 20

....|....|....|....|

**1084 DGKAHF--PREGVFVSNGTH 1101**

**0490 ..DVT.CR.KSP.Y.G..V. 509**

+ +

### Similar pattern 2:

10 20

....|....|....|....|...

**17 NLTTRTQLPPAYTNSFTRGVYYP 39**

**41 .FSSYHGTK.G.VD.IQK.IQK. 63**

++ + + +

### Similar pattern 3:

10 20 30

....|....|....|....|....|....|....|....

**030 NSFTRGVYYPDKVFRSSVLHSTQDLFLPFFSN---VTW 64**

**398 ..YN.SA.S.G--------.K..----..LHDGYA.S. 423**

+ + +

1. Hepatitis B
2. HBsAg-adw2

### Similar pattern 1:

10

....|....|

**614 DVNCTEVPV 622**

**144 .G...CI.I 15**

+ +

### Similar pattern 2:

10

....|....|....|.

**327 VRFPNITNLCPFGEVF 342**

**168 ...SWLSL.V..VQW. 183**

++ +

1. HBsAg-adr

### Similar pattern 1:

10

....|....|

**613 QDVNCTEVPV 622**

**143 S.G...CI.I 152**

+ +

### Similar pattern 2:

10

....|....|....|.

**327 VRFPNITNLCPFGEVF 342**

**168 ...SWLSL.V..VQW. 183**

++ +

### Similar pattern 3:

10

....|....|..

**595 VSVIT****PGTNTSN 606**

**106 .CPLL...S.TS 117**

+ + ++

1. *Bordetella pertussis* toxin protein

### Similar pattern 1:

10 20 30 40

....|....|....|....|....|....|....|....|...

**150 KSWMESEFRVYSSANNCTFEYVSQPFLMDLEGKQGNFKNLREF 192**

**024 RA.AA..LILVRGYG..PSG.A--.LTY.QAHRPSLRARM..L 64**

++ + + +

### Similar pattern 2:

10

....|....|....|

**614 DVNCTEVPV 622**

**385 .G...CI.I 393**

+ +

### Similar pattern 3:

10 20

....|....|....|....|..

**327 VRFPNITNLCPFGEVF 342**

**409 ...SWLSL.V..VQW. 424**

++ +

1. Measles
2. Hemagglutinin protein

10 20 30 40 50 60 70 80

....|....|....|....|....|....|....|....|....|....|....|....|....|....|....|....|

**500 TNGVGYQPYRVVVLSFELLHAPATVCGPKKSTNLVK---------NKCVNFNFNGLTGTGVLNESNKKFLPFQQFGRDIA 570**

**081 ..SIEH.VKD.LTPL.KIIGDEVGLRT.QRF.D...LISDKIKFL.PDREYD.RD.--.WCI.PPERIK.DYD.YCA.V. 158**

+ + + +++ + ++ + ++ + + + + +

1. Fusion protein

10 20 30

....|....|....|....|....|....|

**1247 CCSCGSCCKFDED--DSEPVLK 1266**

**0518 ..CR.R.N.KG.QVGM.R.G.. 539**

1. Rubella
2. Polyprotein E1/E2

No significant similarity found

1. Mumps
2. Fusion protein

### Similar pattern 1:

10 20 30

....|....|....|....|....|....|....|

**450 NYLYRLFRKSNLKPFER****DISTEIYQAGSTPCNGVE 484**

**433 .LTIS.SQTI.TQ.I--.....LSKVNASLQ.A.K 465**

+ + + + ++ +

### Similar pattern 2:

10 20

....|....|....|....|....|..

**1181 KEIDRLNEVAKNLNESLIDLQELGKY 1206**

**0513 ...R.I.FKTNHI.TISSSVDD.IR. 538**

+ ++ + + +

1. Hemagglutinin/neuraminidase protein

### Similar pattern 1:

10 20 30 40 50 60 70

....|....|....|....|....|....|....|....|....|....|....|....|....|....|

**919 NQKLIANQFNSAIGKIQDSLSSTASALGKLQDVVNQNAQALNTLVKQLSSNFGAISSVLNDILSRLDKVE 988**

**060 .DQGLS..LS.ITD..RE.AAVI...V.VMNQ.IHGVTVS.PL---.IEG.QNQLL.T.AT.CTNRNQ.S 126**

+ ++ + ++ + + + ++ + + + + ++

### Similar pattern 2:

10 20 30 40 50 60 70 80

....|....|....|....|....|....|....|....|....|....|....|....|....|....|....|....|

**293 LDPLSETKCTLKSFTVEKGIYQTSNFRVQPTESIVRFPNITNLCPFG-----------EVFNATRFASVY-AWNRKRISN**

**486 .T.YRHQSGINRN.YFTGALLNS.TT..N..LYVSALN.LKV.A.Y.TQGLFASYTTTTC.QD.GD....CVYIMELA..**

++ + + + + + + +

90

....|....|

**CVADYSVL 368**

**I.GEFQI. 573**

**++ +**

### Similar pattern 3:

10 20

....|....|....|....|....|..

**338 FGEVFNATRFASVY---AWNRKRISNC 361**

**366 .PSY.SSR.VQ.AFLVC...QILVT.. 392**

++ + + ++

### Similar pattern 4:

10 20 30 40 50 60 70 80

....|....|....|....|....|....|....|....|....|....|....|....|....|....|....|....|

**072 GTNGTKRFDNPVLPFNDGVYFASTEKSNIIRGWIFGTTLDSKTQSLLIVNNATNVVIKVCEFQFCNDPFLGVYYHKNNKS**

**333 .VKLARE.FR..N.Y.PCSGPQQELDQRAL.SYFPSYFSSRRV..AFL.CAWNQILVTN..----------LVVPS..QT**

+ + + + + + +++ + ++

90

....|....|.

**WMESEFRV 159**

**L.GA.G.. 410**

**+**

### Similar pattern 5:

10

....|....|

**354 NRKRISNC 361**

**121 ..NQV... 128**

++

1. Hepatitis A
2. VP1 protein

### Similar pattern 1:

10 20 30 40

....|....|....|....|....|....|....|....|.

**208 TPINLVRDLP--QGFSALEPLVDLPIGINITRFQT 240**

**160 ..VG.AV.T.WVEKE...S--I.YKTALGAV..N. 192**

+ + + +

### Similar pattern 2:

10 20 30 40 50 60 70

....|....|....|....|....|....|....|....|....|....|....|....|....|....|....|....

**902 MAYRFNGIGVTQNVLY-ENQKLIANQFNSAIGKIQDSLSSTASALGKLQ--DVVNQNAQALNTLVKQLSSNFGAIS 974**

**156 ..W-.TPV.LAVDTPWV.KESALSIDYKT.L.AVRFNTRR.GNIQIR.PWYSYLYAVSG..DG.GDKTD.T..LV. 230**

+ + + + + + ++ + + + ++ + + + + + + + +

### Similar pattern 3:

10 20

....|....|....|....|....|....

**586 DITPCSFGGVSVITPGTNTSNQ 607**

**220 .K.DST..L..IQIANY.H.DE 241**

+ + ++

### Similar pattern 4:

10 20

....|....|....|....|....|

**037 YYPDKVFRSSVLHSTQDLF 55**

**258 .F.RAPLN.NAML..ESMM 276**

+ + + + +

### Similar pattern 5:

10 20

....|....|....|....|....

**358 ISNCVADYSVLYNSASFSTFKCY 380**

**229 V.IQI.N.N---H.DEYLS.S.. 248**

+ + + + + + +

1. VP3 protein

### Similar pattern 1:

10 20

....|....|....|....|

**318 FRVQPTESIVRFPN 331**

**006 ...ST..NV.NLS. 019**

++

### Similar pattern 2:

10

....|....|....

**886 WTFGAGAALQIPF 898**

**054 ..SIPTL.A.F.. 066**

1. Bacillus Calmette-Guérin
2. Immunogenic protein MPB83

10 20 30 40 50

....|....|....|....|....|....|....|....|....|....|..

**1098 NGTHWFVTQRNFYEPQIITTDNTFVSGNCDVVIGIV---NNTVY 1138**

**0169 D...QTLQGADLT---V.GARDDLMVN.AGL.C.G.HTA.A... 209**

+ + + + + + +

1. Immunogenic protein MPB70

10 20 30

....|....|....|....|....|....|.

**1119 NTFVSGNCDVVIGIV---NNTVY 1138**

**0161 .SLKV..A...C.G.STA.A... 183**

+

1. Immunogenic protein MPB64

### Similar pattern 1:

10 20 30

....|....|....|....|....|....|....|

**26 PAYTNSFTRGVYYPDKVFRSSVLHSTQDLFL 56**

**48 ...NINISLPS....QKSLENYIAQ.R.K.. 78**

+ + + + + +

### Similar pattern 2:

10 20 30 40 50 60

....|....|....|....|....|....|....|....|....|....|....|....|

**891 GAALQIPFAMQMAYRFNGIGVTQNVLYENQKLIANQFNSAIGKIQDSLSSTASA 944**

**038 .Q.C..----..SDPAYN.NISLPSY.PD..SLE.Y----.AQTR.KFL.A.TS 83**

+ ++ + + + + ++

## *Haemophilus influenzae* serotype b (Hib)

## Capsulation protein

10 20 30 40

....|....|....|....|....|....|....|....|....

**1098 NGTHWFVTQRNF-----YEPQIITTDNTFVSGNCDV 1128**

**0094 .L..I.I.HGESNKITSVK.IVRIY.HVITA..AGI 129**

+ + + + + +

## Capsular polysaccharide biosynthesis protein

### Similar pattern 1:

10 20 30 40 50 60 70 80

....|....|....|....|....|....|....|....|....|....|....|....|....|....|....|....|

**501 NGVGYQPYRVVVLSFELLHAPATVCGPKKSTN----------------LVKNKCV---NFNFNGLTGTGVLNESNKKFLP**

**099 .NFD..SSSSIPNEVGVW.KSL..KV..NCS.AWFRIYVGIEKDAGEL.I..IFISEN..D.IY.NNLFYH..D.DT.SL**

+ + + + + +

90 100

....|....|....|....|

**FQQFGRDIADTTDAV 576**

**LSD.KENYIEKCND. 193**

**+ + +**

### Similar pattern 2:

10 20

....|....|....|....|.

**1186 LNEVAKNLNESLI 1198**

**0205 V.SII..I.D.A. 217**

+ + + +

## Poliovirus

## VP1 protein (Sabin 1 strain)

### Similar pattern 1:

10 20 30

....|....|....|....|....|....|....|..

**1114 IITTDNTFVSGNCDVVIGIVNNTVYDPLQ 1142**

**0089 ...V..SASTK.K.KLFTVWKI.YK.TV. 117**

+ + + + +

### Similar pattern 2:

10 20 30 40

....|....|....|....|....|....|....|....|

**567 RDIADTTDAVRDPQTLEILDITPCSFGGVSVITP 600**

**024 ..ALPN.E.SGPAHSK..PAL.AVET.ATNPLV. 57**

+ + + + +

## VP1 protein (Sabin 2 strain)

10 20

....|....|....|....|....|...

**10 LVSSQCVNLTTRTQLPPAYTNSF 32**

**05 MIEGAVEGI.KNALV..TS...L 27**

++ + +

## VP1 protein (Sabin 3 strain)

10

....|....|....|.

**293 LDPLSETKCT 302**

**289 ......KGL. 298**

## *Streptococcus pneumoniae* (PCV10)

## Capsular polysaccharide biosynthesis protein [serotype 19F]

### Similar pattern 1:

10 20 30 40 50 60 70 80

....|....|....|....|....|....|....|....|....|....|....|....|....|....|....|....|

**946 GKLQDVVNQNAQALNTLVKQLSSNFGAISSVLNDILSRL----------DKVEAEVQIDRLITGRLQSLQTYVTQQLIRA**

**062 .EKSGLT..DL..GSS...DYREIILS-QD..EEVV.D.KLDLTPKDLAN.IKVT.PV.TR.VS--V.VSDR.PEEAS.I**

+ + + ++ + +++ + ++ + + ++

90 100 110

....|....|....|....|....|....|

**A----EIRASANLAATKMSECVLGQSKR 1039**

**.NSLR.VA.QKIISI.RV.DVTTLEEA. 166**

+ ++ ++ + +

### Similar pattern 2:

10 20

....|....|....|....|....|

**1218 LGFIAGLIAIVMVTIML 1234**

**0182 I..L..V.GTSVIVLI. 198**

+ + + ++ ++

### Similar pattern 3:

10 20

....|....|....|....|....

**703 NSVAYSNNSIAIPTNFT 719**

**033 S...FAYSTFV.KPE.. 49**

+ ++ ++

### Similar pattern 4:

10

....|....|....|..

**508 YRVVVLSFELL 518**

**082 ..EII..QDV. 92**

++ ++

### Similar pattern 5: 10

....|....|....|

**510 VVVLSFELL 518**

**193 .I..IL... 201**

+

## Capsular polysaccharide biosynthesis protein [serotype 23F]

### Similar pattern 1:

10 20 30 40

....|....|....|....|....|....|....|....|

**946 GKLQDVVNQNAQALNTLVKQLSSNFGAISSVLND 979**

**083 ..VKS.LIE.TL.QEVFE..ILVPWD.FCVEMT. 116**

++ + + + + +

### Similar pattern 2:

10 20

....|....|....|....|....

**454 RLFRKSNLKPFERDISTE 471**

**065 .FLVVA..SNE.Q.LTV. 82**

+ + ++

## Capsular polysaccharide biosynthesis protein [serotype 18C]

### Similar pattern 1:

10 20

....|....|....|....|....|

**1218 LGFIAGLIAIVMVTIML 1234**

**0182 I..L..V.GTSVIVLL. 198**

+ + + ++ ++

### Similar pattern 2:

10 20 30 40 50 60 70 80

....|....|....|....|....|....|....|....|....|....|....|....|....|....|....|....|....|...

**703 NSVAYSNNSIAIPTNFTISVTTEILPVSMTKTSVDCTMYICGDSTECSNLLLQYGSFCTQLNRALTGIAVEQDKNTQEVFAQVK 786**

**033 S...FAYSTFV.KPE..--S..R.YV.NRN.E----------EKSGLT.QD..A..YLVKDY.E---.ILS..V-LE..VSDL. 100**

+ ++ ++ + + + + + + + + + +

### Similar pattern 3:

10 20

....|....|....|....|

**951 VVNQNAQALNTLVK 964**

**067 LT..DL..GSY... 80**

+ + +

### Similar pattern 4:

10

....|....|....|

**510 VVVLSFELL 518**

**193 .I..LL... 201**

+

## Capsular polysaccharide biosynthesis protein [serotype 14]

### Similar pattern 1:

10 20 30 40 50 60 70 80

....|....|....|....|....|....|....|....|....|....|....|....|....|....|....|....|....|...

**703 NSVAYSNNSIAIPTNFTISVTTEILPVSMTKTSVDCTMYICGDSTECSNLLLQYGSFCTQLNRALTGIAVEQDKNTQEVFAQVK 786**

**033 S...FAYSTFV.KPE..--S..R.YV.NRDQ----------.EKSGLT.QD..A..YLVKDY.E---.ILS..V-LE..VSDL. 100**

+ ++ ++ + + + + + + + + + + +

### Similar pattern 2:

10 20 30 40 50 60 70 80

....|....|....|....|....|....|....|....|....|....|....|....|....|....|....|....|

**946 GKLQDVVNQNAQALNTLVKQLSSNFGAISSVLNDILSRL----------DKVEAEVQIDRLITGRLQSLQTYVTQQLIRA**

**62 .EKSGLT..DL..GSY...DYREIILS-QD..EEVV.D.KLDLTPKGLAN.IKVT.PV.TR.VS--V.VNDR.PEEAS.I**

+ + + + + +++ + ++ + + ++

90 100

....|....|....|....|....|...

**A----EIRASANLAATKMSECVLGQSKR 1039**

**.NSLR.VA.QKIISI.RV.DVTTLEEA. 166**

+ ++ ++ + +

### Similar pattern 3:

10 20

....|....|....|....|....|

**1218 LGFIAGLIAIVMVTIML 1234**

**0182 I..L..VSGTSVIVFL. 198**

+ + + ++ +

### Similar pattern 4:

10

....|....|....|..

**508 YRVVVLSFELL 518**

**082 ..EII..QDV. 92**

++ ++

## Capsular polysaccharide biosynthesis protein [serotype 9V]

### Similar pattern 1:

10 20

....|....|....|....|..

**508 YRVVVLSFELLHAPAT 523**

**083 ..EII..QDV.EKV.. 98**

++ ++

### Similar pattern 2:

10 20

....|....|....|....|

**951 VVNQNAQALNTLVK 964**

**068 LT..DL..GSY... 81**

+ + +

### Similar pattern 3:

10 20 30 40 50

....|....|....|....|....|....|....|....|....|....|....

**773 EQDKNTQEVFAQVKQIYK-------TPPIKDFGGFNFSQILPDPSKPS 813**

**004 ..NTIEID..QLF.TLW.RKLMILIVALVTGA.A.AY.TFIVK.EYT. 51**

+ + ++ + + +

## Capsular polysaccharide biosynthesis protein [serotype 7F]

### Similar pattern 1:

10 20

....|....|....|....|....|

**1218 LGFIAGLIAIVMVTIML 1234**

**0182 I..L..V.GTSVIVLL. 198**

+ + + ++ ++

### Similar pattern 2:

10 20 30 40 50 60 70 80

....|....|....|....|....|....|....|....|....|....|....|....|....|....|....|....|

**946 GKLQDVVNQNAQALNTLVKQLSSNFGAISSVLNDILSRL----------DKVEAEVQIDRLITGRLQSLQTYVTQQLIRA**

**062 .EKSGLT..DL..GSY...DYREIILS-QD..EEVV.D.KLDLTPKGLAN.IKVT.PV.TR.VS--V.VNDR.PEEAS.I**

+ + + + + +++ + ++ + + ++

90 100

....|....|....|....|....|...

**A----EIRASANLAATKMSECVLGQSKR 1039**

**.NSLR.VA.QKIISI.RV.DVTTLEEA. 166**

+ ++ ++ + +

### Similar pattern 3:

10 20 30 40 50 60 70 80

....|....|....|....|....|....|....|....|....|....|....|....|....|....|....|....|....|...

**703 NSVAYSNNSIAIPTNFTISVTTEILPVSMTKTSVDCTMYICGDSTECSNLLLQYGSFCTQLNRALTGIAVEQDKNTQEVFAQVK 786**

**033 S...FVYSTFV.KPE..--S..R.YV.NRDQ----------.EKSGLT.QD..A..YLVKDY.E---.ILS..V-LE..VSDL. 100**

+ + ++ + + + + + + + + + + +

### Similar pattern 4:

10

....|....|....|..

**508 YRVVVLSFELL 518**

**082 ..EII..QDV. 92**

++ ++

### Similar pattern 5:

10

....|....|....|

**510 VVVLSFELL 518**

**193 .I..LL... 201**

+

## Capsular polysaccharide biosynthesis protein [serotype 6B]

### Similar pattern 1:

10 20

....|....|....|....|..

**508 YRVVVLSFELLHAPAT 523**

**083 ..EII..QDA.EKV.. 98**

++ +

### Similar pattern 2:

10 20

....|....|....|....|

**951 VVNQNAQALNTLVK 964**

**068 LT..DL..GTY... 81**

+ +

## Capsular polysaccharide biosynthesis protein [serotype 5]

### Similar pattern 1:

10 20 30 40 50 60 70 80

....|....|....|....|....|....|....|....|....|....|....|....|....|....|....|....|....|...

**703 NSVAYSNNSIAIPTNFTISVTTEILPVSMTKTSVDCTMYICGDSTECSNLLLQYGSFCTQLNRALTGIAVEQDKNTQEVFAQVK 786**

**033 S...FAYSTFV.KPE..--S..R.YV..RDQ----------.EKSGLT.QD..A..YL.KDY.E---.ILS..V-LE..VSDL. 100**

+ ++ ++ + + + + + + + + + +

### Similar pattern 2:

10 20

....|....|....|....|....|

**1218 LGFIAGLIAIVMVTIML 1234**

**0182 I..LV...V.NVTVLL. 198**

+ + + ++

### Similar pattern 3:

10

....|....|....|..

**508 YRVVVLSFELL 518**

**082 ..EII..QDV. 92**

++ ++

### Similar pattern 4:

10 20

....|....|....|....|....|....

**567 RDIADTTDAVRDPQTLEILDITP 589**

**083 .E.ILSQ.VLEEVVSDLK..L.. 105**

+ + + + + +

1. Capsular polysaccharide biosynthesis protein [serotype 1]

### Similar pattern 1:

10 20

....|....|....|....|....|

**1218 LGFIAGLIAIVMVTIML 1234**

**0182 I..L..V.GTSVIVLL. 198**

+ + + ++ ++

### Similar pattern 2:

10 20 30 40 50 60 70 80

....|....|....|....|....|....|....|....|....|....|....|....|....|....|....|....|....|...

**703 NSVAYSNNSIAIPTNFTISVTTEILPVSMTKTSVDCTMYICGDSTECSNLLLQYGSFCTQLNRALTGIAVEQDKNTQEVFAQVK 786**

**033 S...FAYSTFV.KPE..--S..R.YV.NRNQ----------.EKPGLT.QD..A.AYLVKDY.E---.VLS..V-LE..ISDL. 100**

+ ++ ++ + + + + ++ + + + + +

### Similar pattern 3:

10 20 30 40 50 60 70 80

...|....|....|....|....|....|....|....|....|....|....|....|....|....|....|....|

**946 GKLQDVVNQNAQALNTLVKQLSSNFGAISSVLNDILSRL----------DKVEAEVQIDRLITGRLQSLQTYVTQQLIRA**

**062 .EKPGLT..DL..GAY...DYREIVLS-QD..EEVI.D.KLDLMPKGLAN.IKVT.PV.TR.VS--V.VSDR.PEEAS.I**

+ + + + +++ + ++ + + ++

90 100 110

....|....|....|....|....|....|

**A----EIRASANLAATKMSECVLGQSKR 1039**

**.NSLR.VA.QKIISI.RV.DVTTLEEA. 166**

+ ++ ++ + +

### Similar pattern 4:

10

....|....|....|..

**508 YRVVVLSFELL 518**

**082 ..EI...QDV. 92**

+ ++

### Similar pattern 5:

10

....|....|....|

**510 VVVLSFELL 518**

**193 .I..LL... 201**

+

## Capsular polysaccharide biosynthesis protein [serotype 4]

### Similar pattern 1:

10 20

....|....|....|....|....|

**1218 LGFIAGLIAIVMVTIML 1234**

**0183 I..L..V.GTSVIVLH. 199**

+ + + ++ +

### Similar pattern 2:

10 20 30 40 50 60 70 80 90 100

....|....|....|....|....|....|....|....|....|....|....|....|....|....|....|....|....|....|....|....|

**951 VVNQNAQALNTLVKQLSSNFGAISSVLNDILSRL----------DKVEAEVQIDRLITGRLQSLQTYVTQQLIRAA----EIRASANLAATKMSECV**

**068 LT..DL..GTY...DYREIILS-QD..EEVV.D.KLDLTPKGLAN.IKVT.PV.TR.VS--I.VNDR.PEEAS.I.NSLR.VA.QKIISI.RV.DVT**

+ + + +++ + + + ++ + ++ ++ +

110

....|....|

**LGQSKR 1039**

**TLEEA. 167**

+

### Similar pattern 3:

10 20 30 40 50

....|....|....|....|....|....|....|....|....|....|....

**773 EQDKNTQEVFAQVKQIYK-------TPPIKDFGGFNFSQILPDPSKPS 813**

**004 ..NTIEID..QL..SLW.RKLMILIVALVTGA.A.AY.TFIVK.EYT. 51**

+ + ++ + + +

### Similar pattern 4:

10

....|....|....|

**510 VVVLSFELL 518**

**194 .I..HL... 202**

+

### Similar pattern 5:

10

....|....|....|..

**508 YRVVVLSFELL 518**

**083 ..EII..QDV. 93**

++ ++

**Supplementary material 3**: Similar regions identified between **Envelope amino-acid sequence** of SARS-Cov2 and investigated vaccines: Measles (a1), Bacillus Calmette-Guérin (b1), *Haemophilus influenzae* serotype b (Hib) (c1) and Poliovirus (d1). Patterns of six and more similar amino acid are highlighted in yellow.

1. Measles
2. Hemagglutinin protein

10 20

....|....|....|....|....|...

**17 VLLFLA****FVVFL-LVTLAILTALRL 39**

**37 ...AVL..M..S.IG.LAIAGI.. 60**

+ + + + +

1. Bacillus Calmette-Guérin (BCG)
2. Immunogenic protein MPB64

### Similar pattern 1:

10 20 30

....|....|....|....|....|....|....|..

**25** **VFLLVTLAILTALRLCAYCCNIVNVSLVKPSFY 57**

**05 I.M...AVV.-------L..S--G.ATAA.KT. 28**

+ + + + +

## *Haemophilus influenzae* serotype b (Hib)

## 1. Capsulation protein

10

....|....|....|....

**03 SFVSEETGTLIVNS 16**

**190 .Y.GQV.N..LLL. 203**

+ + ++

## d- Poliovirus

## VP1 protein (Sabin 1 strain)

### Similar pattern 1:

10

....|....|....|....

**058 VYSRVKNLNSSRV 70**

**308 L.P.OTEI..ABI 320**

+ + + +

**Supplementary material 4**: Similar regions identified between **Glycoprotein Membrane amino-acid sequence** of SARS-Cov2 and investigated vaccines: Rubella (a1) and Mumps (b1, b2). Patterns of six and more similar amino acid are highlighted in yellow.

1. Rubella
2. Polyprotein E1/E2

10 20

....|....|....|....|.

**147 GHLRIAGHHLGRCDI 161**

**343 .T..VGQ..RNAS.V 357**

+ +

1. Mumps
2. Fusion protein

10 20

....|....|....|....|

**080 IAIAMACLVGLMWL93**

**503 ..VCLGG.I.IPA.516**

+ + + +

1. Hemagglutinin/neuraminidase protein

10 20 30 40

....|....|....|....|....|....|....|....|

**Query**  **125 HGTILTRPLLESELVIGAVILRGHLRIAGHHLGR 158**

**Sbjct**  **155 .DFSIGH..NMPSFIPT.TSPN.CT..PSFS..K 188**

+ + +

**Supplementary material 5**: Similar regions identified between **Nucleocapsid amino-acid sequence** of SARS-Cov2 and investigated vaccines: Tetanus (a), Hepatitis A (b1), *Haemophilus influenzae* serotype b (Hib) (c1), Poliovirus (d1), *Streptococcus pneumoniae* (PCV10) (e1,e2). Patterns of six and more similar amino acid are highlighted in yellow.

1. Tetanus toxin protein

10 20 30 40

....|....|....|....|....|....|....|....|....|..

**353 LNKHIDAYKTFPPTEPKK----DKKKKADETQALPQRQKKQ 389**

**003 .EYQV..I.KIIDY.Y.IYSGP..EQI...INN.KNKLEEK 43**

+ ++ + +++

1. Hepatitis A
2. VP1 protein

10 20

....|....|....|....|...

**042 PQGLPNNTASWFTALTQ 58**

**120 .H...-S.LR..FN.F. 135**

+

## *Haemophilus influenzae* serotype b (Hib)

## Capsulation protein

### Similar pattern 1:

10 20 30

....|....|....|....|....|....|....|.

**303 QIAQFAPSASAFFG-MSRIGMEVTPSGTWL 331**

**420 R.ES.RE.KR...SDYI.TELLLRYG...I 449**

+ + + +

### Similar pattern 2:

10 20 30

....|....|....|....|....|....|

**164 GTTLPKGFYAEGSRGGSQASSRSS187**

**165 .KGI.AI..VPTWE..IEQENY..188**

+ + +

## Poliovirus

## VP1 protein (Sabin 1 strain)

### Similar pattern 1:

10 20

....|....|....|....|....|

**193 SSRNSTPGSSR****GTSPARMA211**

**177 T.S.PSIFYTY..A...IS195**

+ + + + ++

1. *Streptococcus pneumoniae* (PCV10)

## Capsular polysaccharide biosynthesis protein [serotype 7F]

10 20 30

....|....|....|....|....|....|...

**197 STPGSSRGTSPARMAGNGGDAALALLL 223**

**172 .S.NIK.N.LIGFL..VI.TSVIV... 198**

+ + + +

## Capsular polysaccharide biosynthesis protein [serotype 1]

10 20 30

....|....|....|....|....|....|...

**197 STPGSSRGTSPARMAGNGGDAALALLL 223**

**172 .S.NIK.N.LIGFL..VI.TSVIV... 198**

+ + + +

**Supplementary material 6**: Similar regions identified between **ORF1ab amino-acid sequence** of SARS-Cov2 and investigated vaccines: *Haemophilus influenzae* serotype b (Hib) (a1,a2) and Poliovirus (b1). Patterns of six and more similar amino acid are highlighted in yellow.

## *Haemophilus influenzae* serotype b (Hib)

1. Capsulation protein

10 20 30 40 50

....|....|....|....|....|....|....|....|....|....|....|...

**2923 KDASGKPVPYCYDTNVLEGSVAYESL---RPDTRYVLMDGSIIQFPNTYL2969**

**0107 .IT.V..IVRI..HVITA.NAGIDR.IAHKIFSQ.DVEA.R..PMGD.FI156**

+ + + + + ++ + + ++

1. Capsular polysaccharide biosynthesis protein

Similar Pattern 1:

10 20 30 40

....|....|....|....|....|....|....|....|..

**4462 FVVKRHTFSNYQHEETIYNLLKDCPAVAKHDFFK 4495**

**0511 .AE..NSLFE.YQYH.FFEI.TQLDKQ.NE..H. 544**

++ + +

### Similar Pattern 2:

10 20 30

....|....|....|....|....|....|....|..

**6092 VQMLSDTLK----NLSDRVVFVLWAHGFE 6116**

**0354 I.RIA.SYPSSHI.FKESP...F.DQ.YD 382**

+ ++ + + +

Similar Pattern 3:

10 20 30 40

....|....|....|....|....|....|....|....|....|...

**3181 CT****FLLNKEMYLKLRSDVLLPLTQYNRYLALYNKYKYFSGA 3220**

**0070 .D.I...KISKT.PF..--.KIAFDCTINGK.NFD.Q.SS 107**

+ ++ ++ + + +

b- Poliovirus

## VP1 protein (Sabin 1 strain)

Similar Pattern 1:

10 20 30

....|....|....|....|....|....|..

**2803 SSEIIGYKAIDGGVTRDIASTDTC 2826**

**0038 .K..PALT.VET.A.NPLVPS..V 61**

++ +

Similar Pattern 2:

10 20 30 40 50 60

....|....|....|....|....|....|....|....|....|....|....|....|....|...

**3207 YLALYNKYKYFSGAMDTTSYREAACC-----HLAKALNDFSNSGSDVLYQPPQTSITSAV 3261**

**0198 .VGIS.A.SH.YDGFSKVPLKDQSAALGDSLYG.AS....GILAVR.VNDHNP.KV..KI 257**

**+ + + ++ + + + + + +**

Similar Pattern 3:

10

....|....|....|....

**3489 TLNDFNLVAMK 3499**

**0233 S....GIL.VR 243**

+ ++ ++

**Supplementary material 7**: Similar patterns identified between **ORF1a amino-acid sequence** of SARS-Cov2 and investigated vaccines: Hepatitis B (a1,a2), Measles (b1) and Mumps (c1, c2); Patterns of six and more similar amino acid are highlighted in yellow.

1. Hepatitis B

## 1.  HBsAg-adw2

10 20 30 40 50 60

....|....|....|....|....|....|....|....|....|....|....|....|....|

**2268 EGYLNSTNVTIATYCTGSIPCSVCLSGLDSLDTYPSLETIQITISSFKWDLTAFGLVAEW2327**

**0101 Q.M.PVCPLIPGSTT.STG..KT.TTPAQGNSMF..CCCTKP.DG----NC.CIPIPSS.156**

+ + + + + + + + + +

## 2. HBsAg-adr

10 20 30 40 50

....|....|....|....|....|....|....|....|....|....|.

**2328 FLAYILF----TRFFYVLGLAAIMQLFFSYFAVHFISNSWLMW 2366**

**0157 AF.RF.WEWASV..SWLSL.VPFV.W.AGLSPTVWL.VI.M.. 0199**

+ ++ + ++ +

1. Measles

## 1. Fusion protein

10 20 30 40 50

....|....|....|....|....|....|....|....|....|....|

**4270 CAFAVDAAKAYKDYLASGGQPITNCVKMLCTHTGTGQAITVTPE 4313**

**0369 ..RTLVSGSFGNRFIL.Q.NL.A..ASI..KCYT..TI.NQD.D 0412**

+ + ++ + +

## c- Mumps

## 1. Fusion protein

10 20 30

....|....|....|....|....|....|....|..

**4267 LSFCAFAVDAAKAYKDYLASGGQPITNCVKMLC4299**

**0353 I.A.V.SPI.GSYMRRFV.LD.TIVA..RSLT.0385**

+ + + ++ + +

## 2. Hemagglutinin/neuraminidase

10 20 30 40

....|....|....|....|....|....|....|....|....|..

**3573 KRTIKGTHHWLLLTILTSLLVLVQSTQWSLFFFLYENAF 3611**

**0400 NQ.LM.AEGRV..-.NNR..YYQR..S.WPYEL...IS. 0437**

+ + + + + +

**Supplementary material 8**: Similar patterns identified between **ORF3a amino-acid sequence** of SARS-Cov2 and investigated vaccines: Tetanus (a), *Corynebacterium diphtheria* (b), Hepatitis B (c1) and Mumps (d1). Patterns of six and more similar amino acid are highlighted in yellow.

1. Tetanus toxin protein

### Similar pattern 1:

10

....|....|....|

**137 NPLLYDANYFL 147**

**378 ...R..TE.Y. 388**

**+**

### Similar pattern 2:

10 20 30 40 50 60

....|....|....|....|....|....|....|....|....|....|....|....|

**020 IKDATPSDFVRATATIPIQASLPFGWLIVGVALLAVFQSASKIITLKKRWQLALSKGVHF 079**

**314 .DSE.ADNLEKT..ALS.---..GIGSVM.I.DG..HHNTEE.VA----QSI...S---- 362**

+ ++ + + +

10 20 30 40 50 60

....|....|....|....|....|....|....|....|....|....|....|....|

**080 VCNLLLLFVTVYSHLLLVA.G.EAPF.YLYALVYFLQSINFVR.IMRLWLCWKCRSKNPL 139**

**363 ----.MVAQAIPLVGE..DIGF-..YNF.ESIINLF.VVHNSYNRSAYSPGH.TQ---.F 414**

++ + + + +++ ++ +

10 20 30 40

....|....|....|....|....|....|....|....|....|..

**140 LY..NYFLCWHTNCYDYCIPYNSVTSS..ITSGDGTTSPI.EHDYQI 186**

**415 .H.G------------.AVSW.T.EDS.IRTGFQGE----.G..IK. 445**

+ + + + +

1. *Corynebacterium diphtheria* toxin protein

### Similar pattern 1:

10 20

....|....|....|....|

**137 NPLLYDANYFLCWHTN 152**

**295 ...FAG...-AA.AV. 309**

### Similar pattern 2:

10 20 30

....|....|....|....|....|....|..

**117 SINFVRIIMRLWLCWK----CRSKNPL 139**

**476 .V.GRK.R..CRAIDGDVTF..P.S.V 502**

+ + + +

1. Hepatitis B
2. HBsAg-adw2

10 20 30

....|....|....|....|....|....|...

**039 ASLPFGWLIVGVALLAVFQSASKIITLKKRWQL 71**

**166 ..VR.S..SLL.PFVQW.VGL.PTVW.SAI.MM 198**

+ + + + +

1. Mumps
2. Hemagglutinin/neuraminidase

10 20 30

....|....|....|....|....|....|....|..

**185 QIGGYTEKWESGVKDCVVLHSYFTSDYYQLYSTQLST 221**

**311 E..N--.Q.RYPA.N.KLTRHHMFCQ.NEAERLS.E. 345**

+ + + + + +

**Supplementary material 9**: Similar patterns identified between **ORF6 amino-acid sequence** of SARS-Cov2 and investigated vaccines: Hepatitis B (a1), *Bordetella pertussis* (b), Measles (c1) and Bacillus Calmette-Guérin (BCG) (d1). Patterns of six and more similar amino acid are highlighted in yellow.

1. Hepatitis B
2. HBsAg-adr

10 20

....|....|....|....|....

**024 VRGTTVLLKEPCSSGT—YEGNSPF 46**

**110 IP.S.TTSTG..KTC.TPAQ...M. 134**

+ + + +

1. *Bordetella pertussis* toxin protein

10 20

....|....|....|....|....|...

**41 EGNSPF--HPLADNKFALTCFSTQFAFA 66**

**65 S..E.WPI.G...GQYLGGKYGGELKR. 92**

+ ++ +

1. Measles
2. Fusion protein

10 20

....|....|....|....|...

**021** **QECVRGTTVLLKEPCSSGTYEGN 43**

**359 ...L..Y.KSCARTLV..SF-.. 380**

+ ++

1. Bacillus Calmette-Guérin (BCG)
2. Immunogenic protein MPB64

10

....|....|.

**100** **IFLIVAAIVFI 110**

**005 ..ML.T.V.LL 15**

**++ + +**

**Supplementary material 10**: Similar patterns identified between **ORF7a amino-acid sequence** of SARS-Cov2 and investigated vaccines: *Bordetella pertussis* (a), Measles (b1), Bacillus Calmette-Guérin (BCG) (c1) and Poliovirus (d1). Patterns of six and more similar amino acid are highlighted in yellow.

1. *Bordetella pertussis* toxin protein

10 20 30

....|....|....|....|....|....|..

**41 EGNSPF--HPLADNKFALTCFSTQFAFA66**

**65 S..E.WPI.G...GQYLGGKYGGELKR.92**

+ ++ + +

1. Measles

1. Fusion protein

10 20

....|....|....|....|....|....

**021 QECVRGTTVLLKEPCSSGTYEGN 043**

**359 ...L..Y.KSCARTLV..SF-.. 380**

**+ ++**

1. Bacillus Calmette-Guérin
2. Immunogenic protein MPB64

10

....|....|.

**100IFLIVAAIVFI 110**

**005..ML.T.V.LL 015**

++ + +

1. Poliovirus
2. VP1 protein (Sabin 3 strain)

            10

   ....|....|....|.

**72 KHVYQLRARSVS 83**

**62 R..V.R.S..E. 73**

   +      +

**Supplementary material 11**: Similar patterns identified between **ORF7b amino-acid sequence** of SARS-Cov2 and investigated vaccines: Measles (a1), Rubella (b1) and Mumps (c1), Patterns of six and more similar amino acid are highlighted in yellow and a pattern of ten is highlighted in cyan.

1. Measles
2. Hemagglutinin protein

10

....|....|....|.

**002** **IELSLIDFYL 11**

**201 MS...L.L.. 210**

+ +

1. Rubella
2. Polyprotein E1/E2

10

....|....|....|....

**029 WFSLELQDHNETC 41**

**812 .A.PVC.R.SPD. 824**

**+**

1. Mumps
2. Fusion protein

10

....|....|.

**006** **LIDFY 10**

**286 V.... 290**

+

**Supplementary material 12**: Similar patterns identified between **ORF8 amino-acid sequence** of SARS-Cov2 and investigated vaccines: Measles (a1,a2), Mumps (b1) and Hepatitis A (c1). Patterns of six and more similar amino acid are highlighted in yellow.

1. Measles
2. Hemagglutinin protein

10 20

....|....|....|....|....|..

**55 APLIELCVDEAGSKSPIQYIDI 76**

93 **T..FKIIG..V.LRT.QRFT.L 114**

**++ ++ + +**

1. Fusion protein

10 20 30 40 50

....|....|....|....|....|....|....|....|....|....|...

**031 YVVDDPCPIHFYSKWY****IRVGARKSAPLIELCVDEAGSKSPIQYIDIG 077**

**417 .IAA.H..VVEVNGVT.Q..S.RYPDAVY.HRIDL.PPISLERL.V. 463**

+ + + + + + + + ++ + +

1. Mumps
2. Hemagglutinin protein

10

....|....|....|.

**063 DEAGSKSPIQ72**

**267 .Y...SP.T.276**

1. Hepatitis A
2. VP1 protein

10 20 30 40

....|....|....|....|....|....|....|....|....|....

**030 PYVVDDPCPIHFYSKWYIRVGARKS-APLIELCVDEAGSKSPI 71**

**174 .WIS.T.YRVNR.T.SAHQK.EYTAIGK..VY.YNRLT.P.NV 216**

++ ++ + + + + +

**Supplementary material 13**: Similar patterns identified between **ORF10 amino-acid sequence** of SARS-Cov2 and Bacillus Calmette-Guérin (a1) vaccine.

1. Bacillus Calmette-Guérin

1. Immunogenic protein MPB64

....|

**25 NYIAQ 29**

**68 ..... 72**
