# Supplementary material for: In silico comparative study of SARS-CoV-2 proteins and antigenic proteins in BCG, OPV, MMR and other vaccines: evidence of a possible putative protective effect
Source: BMC Bioinformatics. 2021 Mar 26;22:163. doi: 10.1186/s12859-021-04045-3 (PMC7995392; doi:10.1186/s12859-021-04045-3)
Supplement: Supplementary file 4 — Additional file 4: List of the HLA alleles used for the prediction of CMH-I binding using IEDB analysis resource. [file 12859_2021_4045_MOESM4_ESM.docx]

**Title:**

***In silico* comparative study of** **SARS-CoV-2 proteins and antigenic proteins in BCG, OPV, MMR and other vaccines: evidence of possible putative protective effect**

**Authors:** Sondes Haddad-Boubaker^1,2^, Houcemeddine Othman^3^, Rabeb Touati^4^, Kaouther Ayouni^1, 2^, Marwa Lakhal^4^, Imen Ben Mustapha^5^, Kais Ghedira^6^, Maher Kharrat^4^ and Henda Triki^1,2^

1 Laboratory of Clinical Virology, WHO Regional Reference Laboratory for Poliomyelitis and Measles for EMRO region, Institut Pasteur de Tunis, University of Tunis El Manar, Tunisia.

2. Laboratory of Virus, Host and vectors, Institut Pasteur de Tunis, University of Tunis El Manar, Tunisia.

3 Sydney Brenner Institute for Molecular Bioscience, University of the Witwatersrand, Johannesburg, South Africa.

4 LR99ES10 Human Genetics Laboratory, Faculty of Medicine of Tunis (FMT), University of Tunis El Manar, Tunisia.

5. Laboratory of Transmission, Control and Immunobiology of Infections. Institut Pasteur de Tunis, University of Tunis El Manar, Tunisia

6. Laboratory of Biomathematics, Biomathematics and Biostatistics. Institut Pasteur de Tunis, University of Tunis El Manar, Tunisia.

**Supplementary material 15:** List of the HLA alleles used for the prediction of CMH-I binding using IEDB analysis resource.

HLA-A*01:01

HLA-A*01:01

HLA-A*01:01

HLA-A*01:01

HLA-A*02:01

HLA-A*02:01

HLA-A*02:01

HLA-A*02:01

HLA-A*02:03

HLA-A*02:03

HLA-A*02:03

HLA-A*02:03

HLA-A*02:06

HLA-A*02:06

HLA-A*02:06

HLA-A*02:06

HLA-A*03:01

HLA-A*03:01

HLA-A*03:01

HLA-A*03:01

HLA-A*11:01

HLA-A*11:01

HLA-A*11:01

HLA-A*11:01

HLA-A*23:01

HLA-A*23:01

HLA-A*23:01

HLA-A*23:01

HLA-A*24:02

HLA-A*24:02

HLA-A*24:02

HLA-A*24:02

HLA-A*26:01

HLA-A*26:01

HLA-A*26:01

HLA-A*26:01

HLA-A*30:01

HLA-A*30:01

HLA-A*30:01

HLA-A*30:01

HLA-A*30:02

HLA-A*30:02

HLA-A*30:02

HLA-A*30:02

HLA-A*31:01

HLA-A*31:01

HLA-A*31:01

HLA-A*31:01

HLA-A*32:01

HLA-A*32:01

HLA-A*32:01

HLA-A*32:01

HLA-A*33:01

HLA-A*33:01

HLA-A*33:01

HLA-A*33:01

HLA-A*68:01

HLA-A*68:01

HLA-A*68:01

HLA-A*68:01

HLA-A*68:02

HLA-A*68:02

HLA-A*68:02

HLA-A*68:02

HLA-B*07:02

HLA-B*07:02

HLA-B*07:02

HLA-B*07:02

HLA-B*08:01

HLA-B*08:01

HLA-B*08:01

HLA-B*08:01

HLA-B*15:01

HLA-B*15:01

HLA-B*15:01

HLA-B*15:01

HLA-B*35:01

HLA-B*35:01

HLA-B*35:01

HLA-B*35:01

HLA-B*40:01

HLA-B*40:01

HLA-B*40:01

HLA-B*40:01

HLA-B*44:02

HLA-B*44:02

HLA-B*44:02

HLA-B*44:02

HLA-B*44:03

HLA-B*44:03

HLA-B*44:03

HLA-B*44:03

HLA-B*51:01

HLA-B*51:01

HLA-B*51:01

HLA-B*51:01

HLA-B*53:01

HLA-B*53:01

HLA-B*53:01

HLA-B*53:01

HLA-B*57:01

HLA-B*57:01

HLA-B*57:01

HLA-B*57:01

HLA-B*58:01

HLA-B*58:01

HLA-B*58:01

HLA-B*58:01

HLA-C*01:02

HLA-C*02:02

HLA-C*02:09

HLA-C*03:02

HLA-C*03:03

HLA-C*03:04

HLA-C*04:01

HLA-C*05:01

HLA-C*06:02

HLA-C*07:01

HLA-C*07:02

HLA-C*07:04

HLA-C*08:01

HLA-C*12:02

HLA-C*12:03

HLA-C*14:02

HLA-C*15:02

HLA-C*16:01

HLA-C*17:01

HLA-E*01:01

HLA-E*01:03

HLA-G*01:01

HLA-G*01:02

HLA-G*01:03

HLA-G*01:04

HLA-G*01:06
